# Intermediate Term Results of a Novel Minimally Invasive Keratoprosthesis

**DOI:** 10.1016/j.xops.2026.101117

**Published:** 2026-02-18

**Authors:** Jyoti Sharma, Thomas H. Dohlman, Chengxin Zhou, Elise Taniguchi, Ali Albalakhi, Tharun Somasunder, Peter York, Fengyang Lei, Jie Liu, Robert J. Wood, James Chodosh, Roberto Pineda, Eleftherios I. Paschalis

**Affiliations:** 1 Massachusetts Eye and Ear, Harvard Medical School, Boston, Massachusetts; 2 John A. Paulson School of Engineering and Appllied Sciences, Harvard University, Allston, Massachusetts; 3 Department of Ophthalmology and Visual Sciences, University of New Mexico School of Medicine, Albuquerque, New Mexico

**Keywords:** Cornea, Keratoprosthesis, Implant, Nitinol, Transplantation

## Abstract

**Purpose:**

Keratoprosthesis implantation remains a sight-restoring option in high-risk corneal transplantation where conventional keratoplasty is unlikely to succeed. Existing devices, such as the Boston keratoprosthesis (B-KPro), are very effective but long-term results are limited by glaucoma, infections, retroprosthetic membrane (RPM) formation, and retinal complications. We developed a minimally invasive keratoprosthesis (mi-KPro) that couples an ultrathin nitinol backplate with a poly(methyl methacrylate) optic and is implanted with a modified deep anterior lamellar keratoplasty (DALK) technique.

**Design:**

An experimental study in rabbits.

**Subjects:**

Thirty New Zealand white rabbits.

**Methods:**

Rabbits received severe alkali or acid corneal burns and 1-month later treated with mi-KPro or penetrating keratoplasty (PKP); an additional cohort underwent B-KPro implantation in naïve eyes. Animals were followed for up to 12 months with serial photography, intraocular pressure (IOP) measurements by intracameral manometry and TonoPen, and anterior and posterior segment OCT. Explanted eyes were histologically analyzed.

**Main Outcome Measures:**

Endpoints included device anatomic retention, RPM formation, infection, angle status, retinal thickness, and optic nerve axon density on histology.

**Results:**

The modified DALK approach allowed reproducible mi-KPro implantation with the nitinol backplate seated over Descemet membrane, and the optic extending into the anterior chamber through a 2.5-mm trephination. The mi-KPro eyes maintained clear optics without RPM, haze, or neovascularization and showed stable IOP (15.5 ± 2.2 at last follow-up vs. 15.6 ± 1.5 mmHg at baseline; *P* = 0.138). In contrast, all PKP grafts failed within 2.2 ± 0.7 months with marked corneal edema, neovascularization, and sustained IOP elevation despite timolol. The B-KPro eyes developed early, refractory IOP rise and staphyloma. Tonopen readings strongly correlated with intracameral IOP (*r* = 0.95, *P* = 0.001). OCT and histology demonstrated preservation of angle anatomy, retinal thickness, and optic nerve axons in mi-KPro eyes, whereas PKP eyes exhibited significant retinal thinning (*P* = 0.0014) and axon loss (*P* = 0.0001).

**Conclusions:**

The mi-KPro combines a minimally invasive surgical approach with a flexible nitinol backplate to achieve long-term retention, stable IOP, and preservation of posterior segment structure in severe chemical injury. These data support mi-KPro as a promising next-generation keratoprosthesis for high-risk corneal blindness.

**Financial Disclosure(s):**

Proprietary or commercial disclosure may be found in the Footnotes and Disclosures at the end of this article.

Corneal blindness, a debilitating outcome of severe ocular trauma, among other etiologies, affects approximately 10 million individuals annually worldwide.^1^ While corneal transplantation remains the standard treatment, many patients are ineligible due to severe ocular surface disease, prior repeated graft failures, or donor tissue shortages.^2^^,^^3^ Artificial corneas, or keratoprostheses (KPros), have emerged as a critical alternative, with the Boston keratoprosthesis (B-KPro)—the only US Food and Drug Administration and European Union-approved device—being highly successful in restoring vision in >20 000 eyes globally to date.^4^ Despite advances in KPro technology, including designs like CorNeat,^5^ KeraClear,^6^ KoBra,^7^ and Gore,^8^ long-term success is limited by severe postoperative complications such as glaucoma, retroprosthetic membrane (RPM) formation, retinal detachment, and infectious endophthalmitis.^9^, ^10^, ^11^, ^12^ These challenges necessitate innovative approaches to device design, materials, and surgical techniques to reduce trauma and inflammation.

Here, we introduce a minimally invasive keratoprosthesis (mi-KPro) that addresses existing limitations through a refined architecture. The mi-KPro features a 70-μm super-flexible nitinol backplate, a nickel–titanium alloy widely utilized in biomedical applications such as cardiac stents^13^ and glaucoma trabecular micro-bypass devices^14^ due to its excellent biocompatibility, superelasticity, and shape memory properties.^15^ The poly(methyl methacrylate) optical stem is assembled with the Nitinol backplate using a titanium locking sleeve. We employed finite element analysis to optimize the mechanical behavior of the backplate for uniform stress distribution and improved deformation characteristics to enable conformation to corneal curvature and allow physiological micro-movements for tissue remodeling.^10^, ^11^, ^12^^,^^16^ It was designed to permit intraocular pressure (IOP) measurement with standard tonometers—a critical unmet gap in use of existing KPros. Implantation leverages a modified deep anterior lamellar keratoplasty (DALK) procedure, requiring only a 2.5-mm trephination of Descemet’s membrane, markedly reducing surgical trauma compared to penetrating keratoplasty (PKP) or B-KPro surgery.

This study evaluates the long-term safety and efficacy of the mi-KPro in rabbit models of corneal alkali and acid injuries, benchmarking its performance against PKP and the gold standard of keratoprosthesis, the B-KPro. Our results highlight excellent device retention, infection control, IOP stability, and retinal health, positioning the mi-KPro as a transformative solution for corneal blindness.

## Methods

### Animals

All animal experiments complied with the Association for Research in Vision and Ophthalmology Statement for the Use of Animals in Ophthalmic and Vision Research and the study adhered to the Declaration of Helsinki. The study protocol was approved by the Animal Care Committee of Schepens Eye Research Institute and reviewed by the Animal Care and Use Review Office of the US Department of Defense. Thirty New Zealand white rabbits (aged 3–6 months) were used, with 12 assigned to an alkali injury group, 12 to an acid injury group, and 6 naïve animals as a predicate B-KPro surgery group. Animals were sourced from Charles River Laboratories. General anesthesia was induced via intramuscular injection of xylazine (5–10 mg/kg) and ketamine (30–50 mg/kg), maintained with isoflurane gas as required, and reversed with intravenous yohimbine (0.1 mg/kg). Postprocedure, rabbits were monitored until sternal recumbency and responsiveness were regained, with daily observations for 21 days. Euthanasia was performed under anesthesia by intravenous administration of pentobarbital (100 mg/kg; Fatal-Plus, Vortech Pharmaceuticals).

### Chemical Injury Models

Severe chemical injuries were induced in the right eyes of anesthetized rabbits as follows. Topical proparacaine (0.5%; Alcaine, Alcon) was applied prior to injury. An 8-mm filter paper disc, saturated with either 2N NaOH or 2N HCl (n = 12 per group), was placed on the cornea for 20 seconds, as established in prior studies.^17^, ^18^, ^19^ Postinjury, eyes were irrigated with sterile saline for 15 minutes. Pain was managed with a transdermal fentanyl patch (12 μg, 72 h; Mylan Pharmaceuticals). Postinjury care included ofloxacin 0.3% eye drops (Bausch & Lomb) twice daily for 7 days and prednisolone acetate 1% (Pred Forte, Sandoz) once daily for 21 days.

### Mi-KPro Design, Simulations, and Surgical Implantation

**Figure 1:**
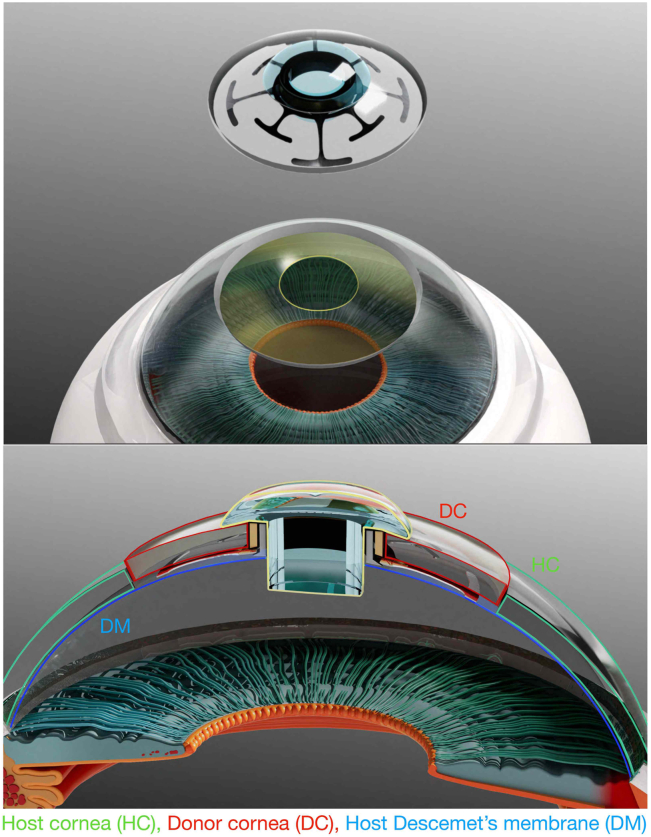
A 3D illustration of mi-KPro preassembled mi-KPro with donor cornea placement over the corneal bed prepared with DALK with central 2.5 mm trephination (host cornea-green outline). Assembled mi-KPro with DC (red outline) sandwiched between nitinol backplate with angled locking and shoulder rings and poly(methyl methacrylate) optics (yellow outline) is placed over the host residual DM (blue). 3D = 3-dimensional; DALK = deep anterior lamellar keratoplasty; DC = donor cornea; DM = Descemet membrane; HC = host cornea; mi-KPro = minimally invasive keratoprosthesis.

**Figure 2:**
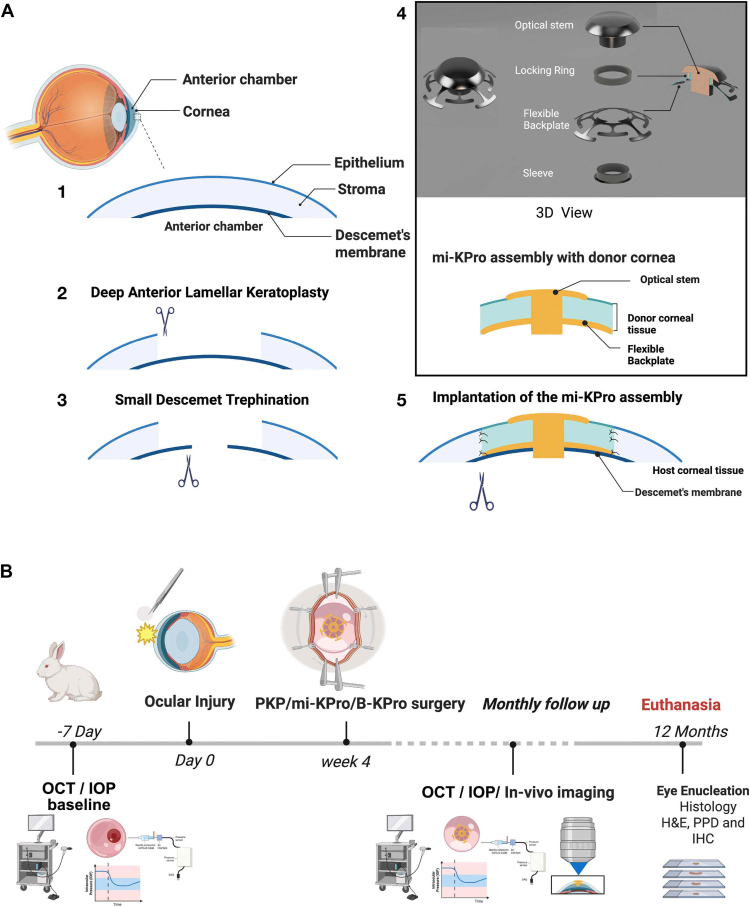
Minimally invasive keratoprosthesis surgical approach and evaluation. (**A**) Graphical representation of the novel surgical procedure to implant KPro: The cornea (1) is partially trephined using Barron trephine (∼7-9 mm), followed by deep anterior lamellar keratoplasty (DALK) procedure (2). Using a small diameter trephine (SDT) (2.5 mm), central Descemet membrane (and residual corneal stroma) is removed, giving access to the anterior chamber (3). Different components of mi-KPro (4) are preassembled with donor cornea and placed on the eye, and the optical stem is inserted by ∼1.5 mm inside the anterior chamber (5). The mi-KPro backplate is positioned over the Descemet membrane (and residual stroma), and the donor and host corneal tissues are sutured together, as in penetrating keratoplasty/B-KPro. (**B**) Timeline of the study design. 3D = 3-dimensional; B-KPro = Boston keratoprosthesis; H&E = hematoxylin and eosin; IHC = immunohistochemistry; IOP = intraocular pressure; mi-KPro = minimally invasive keratoprosthesis; PKP = penetrating keratoplasty; PPD = paraphenylenediamine.

The mi-KPro comprised a medical-grade nitinol (Ti-Ni) backplate, titanium locking sleeve, and poly(methyl methacrylate) optical stem, used with a donor corneal graft carrier (Figure 1, Figure 2B, Video 1, available at www.ophthalmologyscience.org). Static stress analysis was performed in Autodesk Fusion 360 (v2601.0.90) using a linear elastic, isotropic nitinol model (ρ = 4.433 × 10^-6^ kg/mm^3^, E = 50 000 MPa, ν = 0.35, yield = 550 MPa, ultimate tensile strength = 1100 MPa). The central hole was fixed in X/Y/Z, and a bending load derived from IOP was applied: F = P×A = 2066 Pa × 0.0001083 m^2^ = 0.224 N (*P* = 15.5 mmHg; A = circular area, 11.75 mm diameter). A parabolic mesh (2694 elements; 6259 nodes) was used (average element size = 10% of model size; contact tolerance = 0.10 mm), with no rigid body mode removal or adaptive refinement. Safety factor was calculated relative to yield strength.

Surgical implantation occurred 1-month post–chemical injury in the injured right eyes. The procedure included a DALK with a 2.5-mm central Descemet trephination to position the optical stem in the anterior chamber, securing the backplate anterior to Descemet membrane (Figure 1, Figure 2A). Anesthetized rabbits were positioned laterally under a surgical microscope (Leica M844, Leica Microsystems). Topical proparacaine (0.5%) was administered. The surgical field was prepared with 10% povidone–iodine (Betadine, Purdue Products) to the eyelids, with 5% povidone–iodine instilled onto the ocular surface. An eyelid speculum was placed, and a Barron trephine (Katena Products) used to trephine the cornea to two-thirds depth, guided by OCT. The anterior stroma was separated from Descemet membrane using irrigation with a 30G needle and dissected with a crescent blade. Central trephination (2.5 mm) was performed with a biopsy punch through which the elongated poly(methyl methacrylate) optical stem was advanced into the anterior chamber through the 2.5 mm Descemet’s opening, followed by limited injection of viscoelastic (Healon, Johnson & Johnson Vision) into the anterior chamber. The mi-KPro, assembled with a fresh rabbit donor cornea (Pel-Freez Biologicals), was sutured with 10-0 nylon (Ethicon) in interrupted fashion. Every animal received ≥16 sutures, as required, which were removed during follow-ups if they became loose. A partial temporary eyelid tarsorrhaphy was performed using 6-0 Vicryl (Ethicon) sutures and maintained for 7 days. Animals were kept on a heating pad with vital signs monitored. Postsurgery, subcutaneous lactated Ringer’s solution (20 ml) and yohimbine were administered. Operated eyes received triple antibiotic ointment (Bausch & Lomb) once daily for 7 days, ofloxacin 0.3% twice daily for 7 days, and Pred Forte 1% once daily for 21 days.

### PKP

Penetrating keratoplasty was performed as a surgical control using an 8-mm trephine (Katena Products) to excise the host cornea, followed by suturing a fresh donor cornea (Pel-Freez Biologicals) with 10-0 nylon interrupted sutures. The tarsorrhaphy and postoperative care mirrored the mi-KPro protocol.

### B-KPro

A separate study was performed by our group to assess the retention of predicate B-KPro in rabbit eyes (device implantation reference). Human size B-KPros were implanted in 6 New Zealand white rabbits and evaluated for anatomic retention and IOP fluctuation. Briefly, an autologous 8.5 mm diameter corneal graft was used for KPro assembly. The optical cylinder was inserted through a 3 mm in diameter central trephination of the graft, and the back plate was snapped in place behind graft. The KPro was implanted using sixteen 10-0 nylon interrupted sutures (Ethicon), and a temporary partial tarsorrhaphy was applied for 1 week for corneal protection using 6-0 monofilament nylon sutures. Ketoprofen was administered once daily at a dose of 3 mg/kg subcutaneously for 2 days. Ofloxacin eye drops were administered topically twice daily for 21 days to reduce the risk of bacterial contamination.

### Photography and *In Vivo* OCT

High-resolution digital photography of anesthetized animals was conducted along with OCT imaging postinjury and at all surgical follow-up points (Fig 2B). Anterior and posterior segment OCT was performed using a Heidelberg Spectralis system (Heidelberg Engineering GmbH) on anesthetized rabbits. Topical proparacaine (0.5%) and an eyelid speculum maintained ocular access, with sterile saline applied for hydration. Imaging of the cornea, angle, retina, and optic nerve occurred at baseline and monthly for 12 months. Quantification was performed with ImageJ (National Institutes of Health), measuring central and peripheral corneal thickness, retinal thickness (ganglion cell layer to outer nuclear layer), angle parameters (500 μm from trabecular-iris space area,^20^ and optic^21^ nerve cup diameter and depth.

### IOP Measurements

Anesthetized rabbits received topical proparacaine (0.5%), and IOP was measured 5–7 minutes after induction with a TonoPen XL (Medtronic), followed by intracameral recording using a 27G needle connected to a digital pressure sensor.^22^ Ofloxacin 0.3% was applied postmeasurement. Intraocular pressure was recorded at baseline and monthly for 12 months (Fig 2B); pressures >21 mmHg were managed with antiglaucoma medication twice a day until normalized.

### Histopathology of Enucleated Eyes

Enucleated eyes were fixed in 4% paraformaldehyde (Sigma-Aldrich) for 72 hours, and then dissected to isolate the cornea, retina, and optic nerve. Optic nerves were further fixed in half-strength Karnovsky solution (2% paraformaldehyde, 2.5% glutaraldehyde in 0.1M sodium cacodylate buffer, pH 7.4; Electron Microscopy Sciences) and stained with paraphenylenediamine. Corneas and retinas were stained with hematoxylin and eosin. Explanted devices were preserved in half-strength Karnovsky fixative for scanning electron microscopy. Scanning electron microscopy samples were rinsed in 0.1M sodium cacodylate buffer, post fixed in 2% osmium tetroxide, dehydrated in an ethanol gradient (25%–100%), and critical-point dried (SAMDRI-795, Tousimis). Samples were mounted on aluminum stubs, sputter-coated with 10 nm gold (SMARTCoater, JEOL), and imaged at 15 kV under high vacuum (JCM-7000 NeoScope, JEOL).

### Statistical Analyses

Data were analyzed using GraphPad Prism v10.0 (GraphPad Software) and expressed as mean ± standard deviation. Changes in corneal and retinal thickness and optic nerve axon density were compared between groups using unpaired *t* tests, with contralateral eyes as internal controls to reduce variability. Statistical significance was set at *P* < 0.05.

## Results

### Mi-KPro Design Optimization

**Figure 3:**
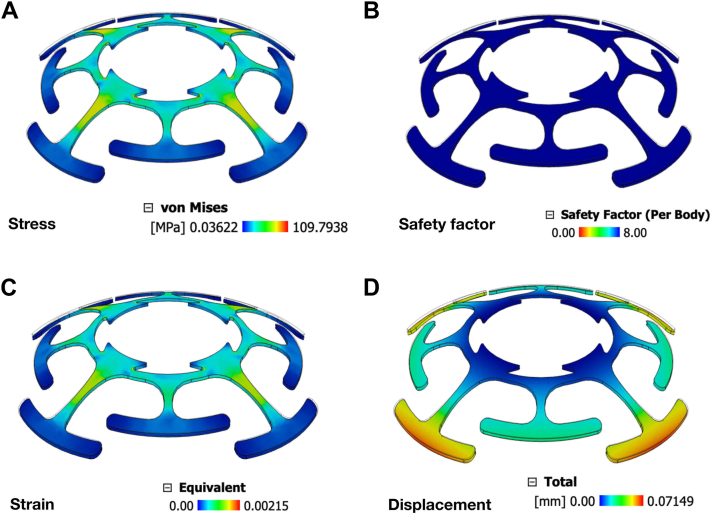
Finite element analysis and optimization of the mi-KPro design. Finite element analysis of the Nitinol backplate with 6259 nodes and 2694 parabolic solid elements. The central hole was fixed in all directions (Ux, Uy, and Uz) and a force of 0.224 N was applied to backplate. (**A**) Stress distribution across the structure. The color gradient represents stress levels, with blue indicating lower stress (minimum: 0.042372 MPa) and red indicating higher stress (maximum: 176.108 MPa). (**B**) Safety factor distribution, showing the ratio of material strength to applied stress. Blue regions indicate a higher safety factor (maximum: 15.00), while red regions indicate a lower safety factor (minimum: 1.0000). (**C**) Strain distribution, illustrating the deformation experienced by the structure. The color scale ranges from blue (minimum strain: 0.0000) to red (maximum strain: 0.001974). (**D**) Displacement distribution, measured in millimeters (mm), showing the physical deformation of the structure under load. Blue regions indicate minimal displacement (minimum: 0.000 mm), while red regions indicate maximum displacement (maximum: 5.57148 mm). mi-KPro = minimally invasive keratoprosthesis.

The superelastic nitinol backplate provided a strong balance of flexibility and strength for dynamic ocular loading. The static stress model preserved geometric detail (contact tolerance 0.10 mm; no rigid-body mode removal; no adaptive refinement; 20% von Mises convergence). Under the applied load, predicted stresses remained below the 550 MPa yield strength with an acceptable yield-based safety factor (Fig 3A, B), indicating no plastic deformation. Although the 3 mm central hole produced a stress concentration, the 1100 MPa ultimate tensile strength provided substantial margin, and no excessive deformation or failures were observed (Fig 3A, C, D). Overall, the design appears mechanically stable for long-term keratoprosthesis implantation.

### Anatomic Retention

#### Surgical Outcomes, Device Retention, and Graft Survival

**Figure 4:**
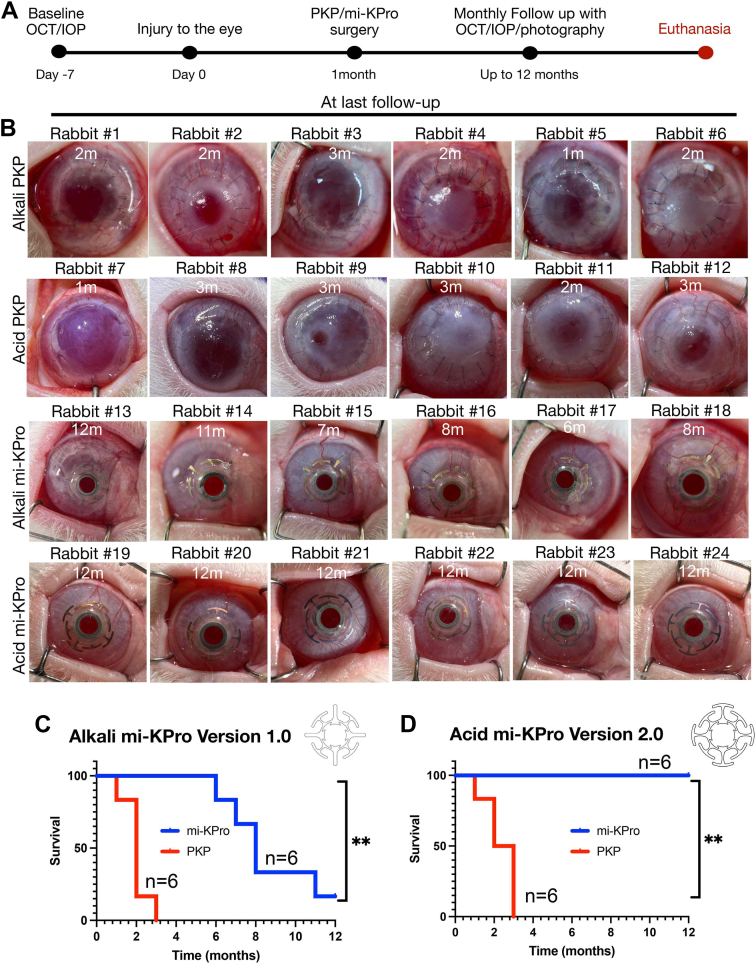
Clinical photographs of rabbit corneas treated with mi-KPro and PKP after alkali or acid burns. (**A**) Timeline of the study. (**B**) Alkali burn eyes treated with PKP exhibit corneal opacity, edema, and neovascularization. Acid burn eyes treated with PKP show corneal thinning, edema, and neovascularization. Alkali burn eyes treated with mi-KPro demonstrate clear optics, device retention for ≥6 months, and corneal melts in 5 animals. Acid burn eyes treated with mi-KPro (version 2) show minimal inflammation, stable device retention, no corneal thinning, clear optics, and 100% retention at 1 year. (**C, D**) Survival curve of mi-KPro and PKP-treated eyes postalkali or acid burns (*P* = 0.0006, n = 12 for alkali; *P* = 0.001, n = 12 for acid). IOP = intraocular pressure; mi-KPro = minimally invasive keratoprosthesis; PKP = penetrating keratoplasty.

**Figure 5:**
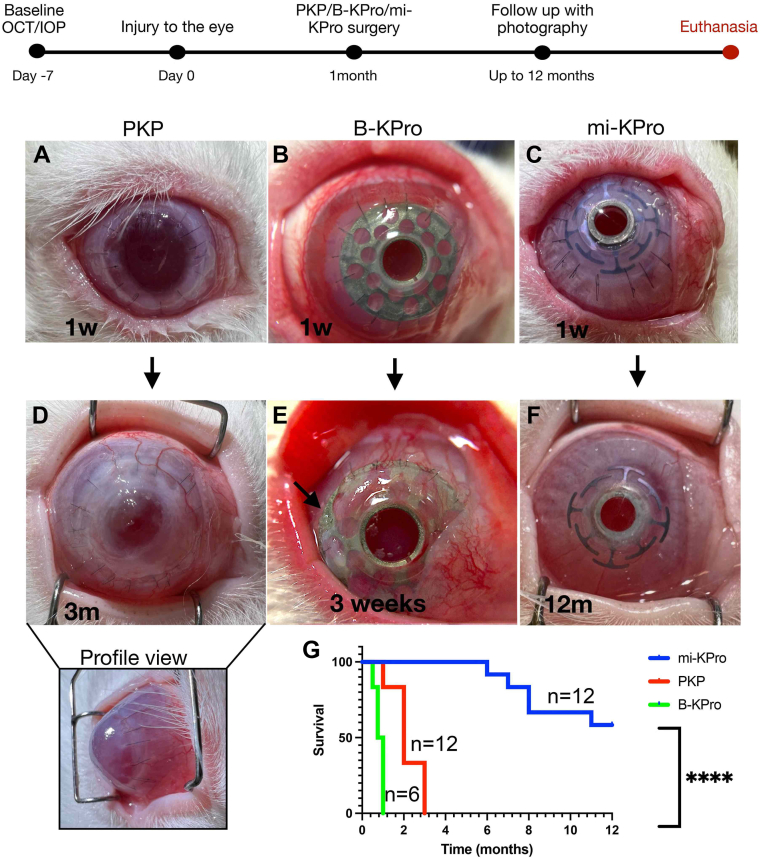
Time-course photographs of rabbit corneas postsurgery. (**A**, **D**) Penetrating keratoplasty at 1 week and 3 months, showing neovascularization, opacification, and graft failure. (**B**, **E**) Boston keratoprosthesis at 1 week and 3 weeks, showing corneal melt and structural compromise of the graft device interface (black arrow). (**C**, **F**) Minimally invasive keratoprosthesis post–acid burn at 1 week and 12 months, with minimal inflammation, no RPM formation, and excellent anatomic retention for 1 year. (**G**) Survival curve of mi-KPro, PKP and B-KPro treated eyes (*P* ≤ 0.0001). B-KPro = Boston keratoprosthesis; IOP = intraocular pressure; mi-KPro = minimally invasive keratoprosthesis; PKP = penetrating keratoplasty; RPM = retroprosthetic membrane.

**Figure 6:**
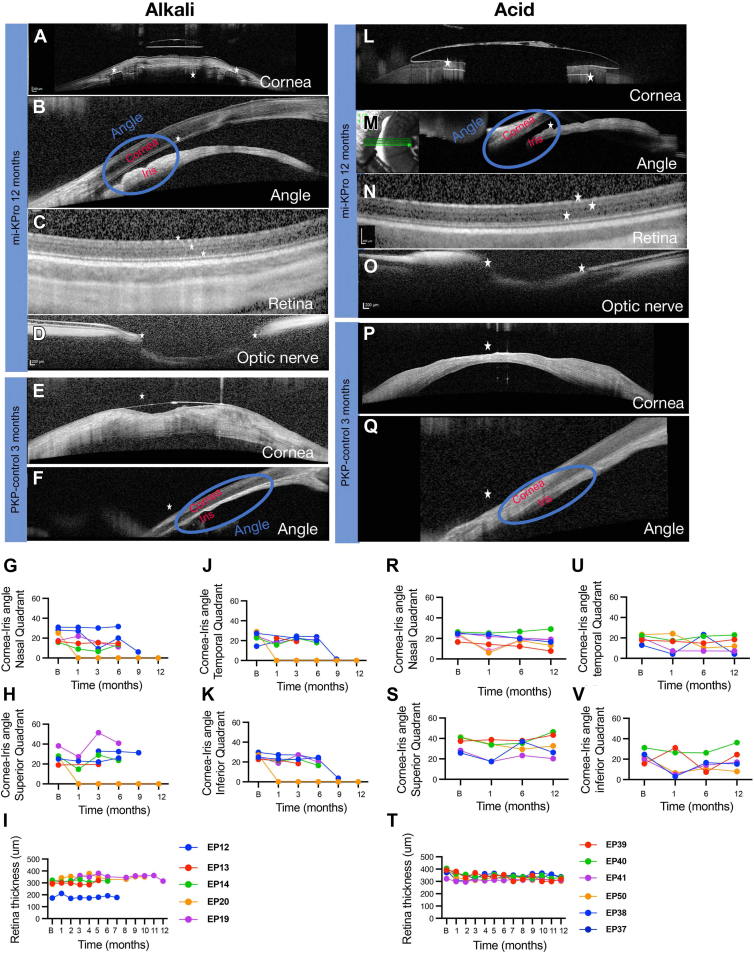
Anterior and posterior OCT post-mi-KPro implantation in alkali and acid burn eyes. (**A**) Corneal OCT showing excellent device retention (asterisk) and donor-host tissue integration in alkali burns. (**B**) Angle OCT indicating an open angle with 35% narrowing. (**C**) Retinal OCT at 12 months revealing intact architecture and minimal thickness reduction (n = 6, *P* = 0.33). (**D**) Optic nerve OCT showing dense nerve fiber tissue and no cupping (asterisk). (**E**) Penetrating keratoplasty-treated eyes exhibit corneal ulcer (asterisk), staphyloma, and opacification. (**F**) Angle OCT in PKP eyes shows complete closure due to 360° iris–corneal adhesion (asterisk). Retinal and optic nerve imaging was not possible due to poor corneal clarity. (**G**–**K**) Graphical representations of all angle and retinal thickness measurements throughout the study in alkali burns. (**I**) Corneal OCT images demonstrating excellent device retention (indicated by an asterisk) and donor-host integration in acid burn eyes. (M) Angle OCT images indicating an open angle with a narrowing of 26%. (**N**) Retinal OCT imaging at 12 months showing intact architecture and minimal thickness reduction (n = 6, *P* = 0.33). (**O**) Optic nerve OCT imaging showing dense nerve fiber tissue and no cupping (indicated by an asterisk). (**P**) Penetrating keratoplasty-treated eyes exhibit corneal ulceration (indicated by an asterisk), staphyloma, and opacification. (**Q**) Angle OCT imaging in PKP eyes showing complete closure due to 360° iris–corneal adhesion (indicated by an asterisk). Imaging of the retinal and optic nerve was not possible due to poor corneal clarity. (**R**–**T**) Graphical representations of all angle and retinal thickness measurements throughout the acid burn study. mi-KPro = minimally invasive keratoprosthesis; PKP = penetrating keratoplasty.

Two models of chemical injury (alkali and acid) were employed to recapitulate clinical scenarios. One-month postinjury, 12 New Zealand white rabbits received cornea replacement with mi-KPro (6 alkali/6 acid burn) and 12 PKP (6 alkali/6 acid burn) (Fig 4A). Two corneal surgeons (RP and THD) with prior experience in PKP and DALK performed the surgeries. The mi-KPro implantation was technically less challenging compared with DALK, because there was no requirement for complete removal of the corneal stroma from the Descemet membrane. Instead, 70–100 μm of residual corneal stroma was left over the Descemet membrane that was trephined along with the central Descemet membrane using a 2.5 mm in diameter tissue biopsy punch. On average, mi-KPro surgery required 45 minutes to 1 hour and PKP 30–45 minutes with standard instruments. All animals were followed for 12 months or until failure, using standard photography, OCT, and IOP measurements (Fig 3B). All procedures were uneventful, and no eyes underwent lensectomy. In 1 mi-KPro surgery, a suture engaged the iris inadvertently causing partial angle closure, which did not lead to failure (Table 1). The mi-KPro devices were anatomically retained with clear optics for 8.6 ± 2.3 (mean ± standard deviation) months in alkali burns and 12 months in acid burns, with combined average retention of 10.3 ± 2.3 months. Postsurgical inflammation was minimal after mi-KPro surgery, and all eyes remained free of infections (Figs 4B–D, 5C, F, 6A, L).

**Table 1:** Outcomes of Rabbits Undergoing mi-KPro Implantation, PKP, or B-KPro

ID	Sex	Injury	Surgical Intervention	Graft Survival (Months)	Infections	RPM Formation	IOP (9-21 mmHg)	Remarks
EP1	F	Naive	B-KPro	21 days	-	-	Hypertensive; Timolol BD	Neovascularization 360, thinning 360
EP2	F	Naive	B-KPro	21 days	-	-	Hypertensive; Timolol BD	Neovascularization 360, thinning 360
EP3	F	Naive	B-KPro	21 days	-	-	Hypertensive; Timolol BD	Neovascularization 360, thinning 360
EP4	M	Naive	B-KPro	21 days	-	-	Hypertensive; Timolol BD	Donor graft failure/thinning
EP5	M	Naive	B-KPro	21 days	-	-	Hypertensive; Timolol BD	Donor graft failure/thinning
EP6	M	Naive	B-KPro	21 days	-	-	Hypertensive; Timolol BD	Donor graft failure/thinning
EP12	M	Alkali	mi-KPro	8	-	No	Normal	Partial graft melt
EP13	M	Alkali	mi-KPro	6	-	No	Normal	Device extrusion
EP14	M	Alkali	mi-KPro	7	-	No	Normal	Partial graft melt
EP18	F	Alkali	mi-KPro	8	-	No	Normal	Partial graft melt
EP19	F	Alkali	mi-KPro	12	-	No	Normal	Normal, device well retained, Angle closure due to intraoperative suture
EP20	F	Alkali	mi-KPro	11	-	No	Normal	Partial exposure at 11 months
EP15	M	Alkali	PKP	2	-	-	Hypertensive; Timolol BD	Cornea with peripheral opacity around the PKP junction
EP16	M	Alkali	PKP	2	-	-	Hypertensive; Timolol BD	Staphyloma/graft failure
EP17	M	Alkali	PKP	3	-	-	Hypertensive; Timolol BD	Staphyloma/graft failure
EP21	F	Alkali	PKP	2	-	-	Hypertensive; Timolol BD	Cornea ulcer/opacification
EP22	F	Alkali	PKP	2	-	-	Hypertensive; Timolol BD	Cornea ulcer/opacification
EP23	F	Alkali	PKP	1	-	-	Hypertensive; Timolol BD Antibiotics	Refractory IOP elevation
EP37	M	Acid	mi-KPro	12	-	No	Normal	No complications
EP38	M	Acid	mi-KPro	12	-	No	Normal	No complications
EP39	M	Acid	mi-KPro	12	-	No	Normal	No complications
EP40	F	Acid	mi-KPro	12	Fungal Natamycin	No	Normal	No complications
EP41	F	Acid	mi-KPro	12	Fungal Natamycin	No	Normal	No complications
EP50	F	Acid	mi-KPro	12	-	No	Normal	No complications
EP32	M	Acid	PKP	1	-	-	Hypertensive; Timolol BD	Refractory IOP elevation
EP34	M	Acid	PKP	3	-	-	Hypertensive; Timolol BD	Staphyloma/graft failure
EP36	M	Acid	PKP	3	-	-	Hypertensive; Timolol BD	Staphyloma/graft failure
EP42	F	Acid	PKP	3	-	-	Hypertensive; Timolol BD	Corneal ulcer/opacification
EP43	F	Acid	PKP	2	-	-	Hypertensive; Timolol BD	Corneal ulcer/opacification
EP49	F	Acid	PKP	1	-	-	Hypertensive; Timolol BD	Staphyloma/graft failure

BD = twice daily; B-KPro = Boston keratoprosthesis; F = female; IOP = intraocular pressure; M = male; mi = micro-incision; mi-KPro = minimally invasive keratoprosthesis; PKP = penetrating keratoplasty; RPM = retroprosthetic membrane.

Rabbit details including injury type, surgical intervention, graft survival duration (in months), presence of infections, RPM formation, IOP, and remarks.

Remarks include complications such as partial graft melt, device extrusion, angle closure, corneal opacity, staphyloma, graft failure, corneal ulceration/opacification, and refractory IOP elevation.

In contrast, all PKP grafts failed within 2.2 ± 0.7 (mean ± standard deviation) months of the surgery from severe neovascularization, opacification, and edema (corneal thickness increased from 396 ± 5.4 μm at baseline to 701.3 ± 111.7 μm, *P* = 0.0035). One animal was euthanized within 1 month due to corneal infection and high IOP. Four of the 5 alkali-burned eyes developed bullous keratopathy (*P* = 0.03), and 3 of 6 acid-burned eyes corneal thinning (*P* = 0.03), with persistent edema and ulceration, which did not improve at 3 months postoperatively (Figs 4B, 5A, D, Supplementary Fig S1, available at www.ophthalmologyscience.org). Predicate control surgery using the B-KPro resulted in failure within 3 weeks of the surgery, primarily due to refractory elevation of the IOP and subsequent staphyloma (Fig 5B, E). Because B-KPro requires large diameter corneal trephination, we hypothesize that the surgical trauma near the limbus contributed to limbal inflammation and angle closure.^23^

Six eyes with alkali burns were initially tested with the mi-KPro device. One eye extruded the device at 6 months, while 4 exhibited partial backplate exposure between 6 and 11 months after surgery. Remarkably, the average anatomic retention rate was 71.6%. Notably, none of these complications resulted in retinal complications, such as detachment. This was because the small 2.5 mm in diameter Descemet membrane trephination self-sealed, and the eye retained its chamber, as evidenced by in vivo photography and postmortem histology of the eyes (Supplementary Fig S2A, available at www.ophthalmologyscience.org). Further analysis revealed increased tissue pressure from the long, thin backplate haptics. The issue was addressed by increasing the contact area of the long haptics in a revised engineering version of the mi-KPro backplate and was tested in acid injuries (Supplementary Fig S2B, available at www.ophthalmologyscience.org). Consequently, the mi-KPro device demonstrated 100% anatomic retention in acid burns for >12 months without tissue thinning. Furthermore, it significantly outperformed the predecessor B-KPro in terms of anatomic retention, ocular inflammation, neovascularization, and stromal edema (Figure 4, Figure 5).

#### Twelve-Month Device Outcomes

Postoperative prophylaxis consisted of daily 0.3% ofloxacin twice daily, triple antibiotic ointment once daily for 1 week, and 1% Pred Forte once daily for 3 weeks, with no further treatment unless clinically warranted. Penetrating keratoplasty eyes showed elevated rates of infection and IOP compared with mi-KPro eyes. One of 6 PKP eyes with alkali burns developed a corneal infection and IOP elevation to 40 mmHg at 1 month, requiring euthanasia.

No infections were noted for the duration of the follow-up, with the exception of 2 mi-KPro eyes with acid burns, which developed suspected fungal infections immediately after surgery, attributed to graft contamination (both eyes were implanted the same day from the same donor tissue container). Despite this, we successfully treated both infections using 5% povidone–iodine (1–2 drops) applied on the cornea for 1 minute, followed by a 0.9% saline flush and 5% natamycin eyedrops 3 times daily for 2 weeks. Remarkably, both eyes remained infection-free for 12 months postoperatively (Supplementary Fig S2C, available at www.ophthalmologyscience.org).

Scanning electron microscopy of the explanted devices revealed no bacterial growth. A thick fibrous tissue layer on the backplate haptics and graft-device interface indicated device-tissue fixation, which is expected to reduce the risk of endophthalmitis (Supplementary Fig S3A, B, available at www.ophthalmologyscience.org).

### Retroprosthetic Membrane Formation

In general, the mi-KPro eyes did not exhibit RPM formation, as evidenced by clear optics throughout the follow-up period (Figs 4, 6C, D, N, O). The elongated optical stem, inserted into the anterior chamber through a small Descemet’s trephination, may contribute to the low rate of RPM formation.

### Posterior Segment OCT and Postmortem Histology

In contrast, PKP and B-KPro eyes developed 360° anterior chamber angle closure within a month, while mi-KPro eyes retained open angles. At 1 month, 30% of alkali-burned and 26% of acid-burned mi-KPro eyes had narrowed angles compared with preoperative measurements, which progressed to 35% and 18%, respectively, at the last follow-up (8.6 ± 2.3 months for alkali-burned eyes and 12 months for acid-burned eyes) (Fig 6B, G–K, Q–V). Notably, 1 alkali-burned mi-KPro eye with partial angle closure was attributed to inadvertent iris suture. Despite this, the mi-KPro eye did not exhibit retinal or optic nerve degeneration (*P* = 0.33; Fig 6C, D, K, N, O, T).

**Figure 7:**
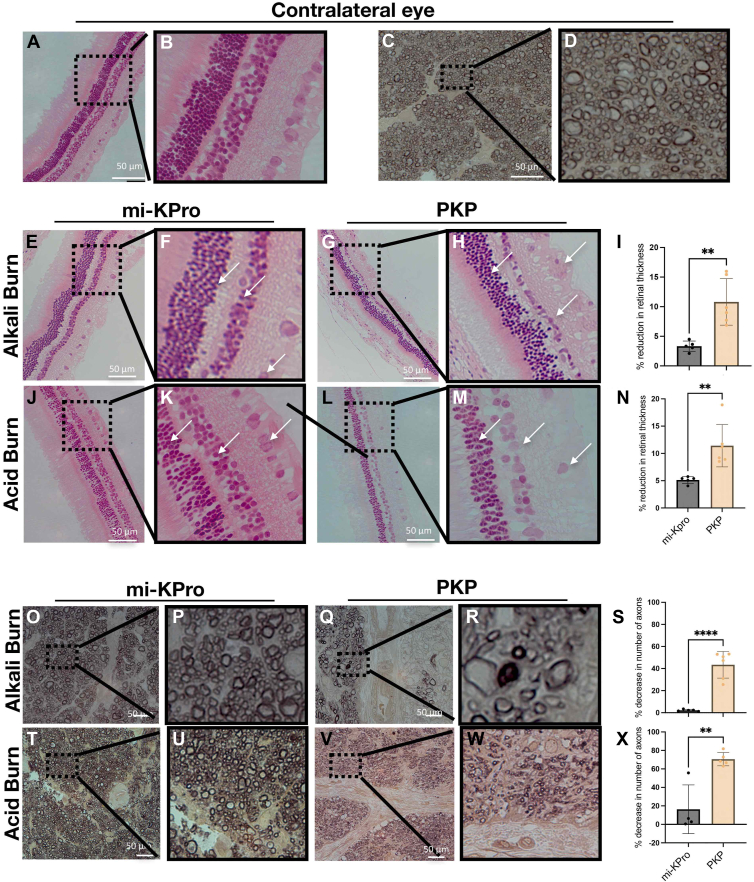
Histology of the retina and optic nerve after surgery. (**A**, **B**) Hematoxylin and eosin (H&E)–stained retinal sections from naïve eye at 10× magnification. (**C**, **D**) Paraphenylenediamine (PPD)-stained optic nerve sections from naïve eye. (**E**, **F**, **J**, **K**) Hematoxylin and eosin (H&E)-stained retinal sections from mi-KPro-treated eyes (alkali and acid burns, respectively) at 10× magnification, showing preserved retinal structure. (**G**, **H**, **L**, **M**) Penetrating keratoplasty-treated eyes exhibit significant retinal degeneration. (**I**, **N**) Quantitative analysis (normalized to contralateral eyes) reveals significantly reduced retinal thickness in PKP-treated eyes compared with mi-KPro (n = 6, *P* = 0.006 for alkali; n = 6, *P* = 0.002 for acid). (**O**, **P**, **T**, **U**) Paraphenylenediamine-stained optic nerve sections from mi-KPro-treated eyes (alkali and acid burns, respectively) at 63× magnification, showing preserved axon density. (**Q**, **R**, **V**, **W**) Penetrating keratoplasty-treated eyes exhibit significant axon loss. (**S**, **X**) Quantitative analysis (normalized to contralateral eyes) reveals significantly reduced axon density in PKP-treated eyes (n = 6, *P* = 0.002 for alkali; n = 6, *P* < 0.0001 for acid). mi-KPro = minimally invasive keratoprosthesis; PKP = penetrating keratoplasty.

**Figure 8:**
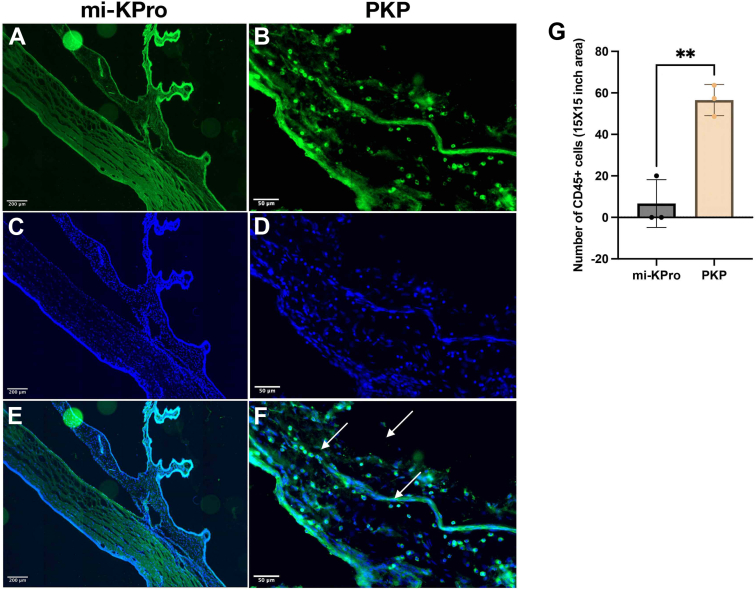
Immune cell infiltration in the cornea–iris angle after mi-KPro and PKP surgery. (**A**, **C**, **E**) Fluorescence microscopy of CD45-stained sections from mi-KPro-treated eyes, showing minimal CD45^+^ immune cell infiltration at the cornea–iris angle. (**B**, **D**, **F**) Penetrating keratoplasty-treated eyes exhibit significant CD45^+^ leukocyte infiltration. (**G**) Quantitative analysis confirms significantly higher CD45^+^ infiltration in PKP eyes (n = 3, *P* = 0.003). mi-KPro = minimally invasive keratoprosthesis; PKP = penetrating keratoplasty.

In contrast, PKP and B-KPro eyes could not be imaged with OCT due to severe corneal stromal edema and RPM, respectively. Histological analysis revealed significant retinal thinning (*P* = 0.006 for alkali; *P* = 0.002 for acid) and optic nerve axon loss (*P* = 0.002 for alkali; *P* = 0.0001 for acid) in PKP eyes compared with mi-KPro. Conversely, the implanted eye in the mi-KPro group exhibited negligible loss in retinal thickness compared with the contralateral naive eye (Fig 7). Using immunohistochemistry, we confirmed that PKP eyes had significantly more infiltration of CD45^+^ cells in the cornea–iris angle compared to mi-KPro eyes (*P* = 0.003; Fig 8).

Hematoxylin and eosin and paraphenylenediamine staining further confirmed that mi-KPro surgery resulted in significantly less retinal thinning (*P* = 0.006 for alkali and *P* = 0.002 for acid) and optic nerve axon loss (*P* = 0.002 for alkali and *P* = 0.0001 for acid) compared with PKP (Fig 7).

### IOP

**Figure 9:**
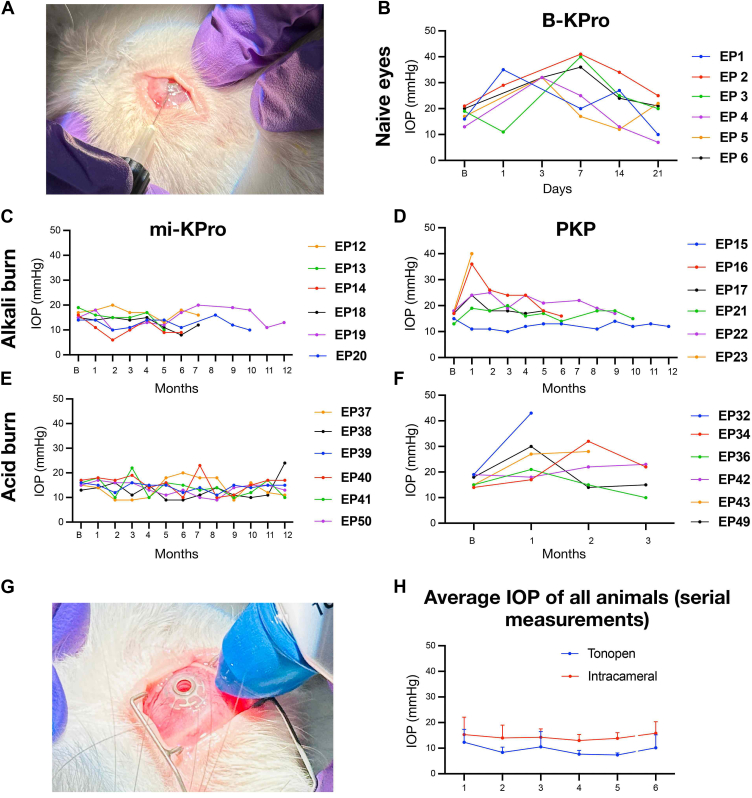
Longitudinal IOP measurements postsurgery: Monthly intracameral IOP measurements in 30 rabbits presurgery and postsurgery. (**A**, **C**, **E**) Minimally invasive keratoprosthesis-treated eyes (n = 12, alkali and acid burns) maintain stable IOP without antiglaucoma medication (*P* = 0.138). (**D**, **F**) Penetrating keratoplasty-treated eyes (n = 12) and (**B**) B-KPro implanted eyes (n = 6) exhibit acute IOP elevation unresponsive to timolol eye drops (*P* = 0.003 and *P* = 0.018, respectively). (**G**, **H**) Monthly IOP trends using TonoPen and intracameral pressure measurements in mi-KPro-treated eyes showing excellent correlation (*r* = 0.95, *P* = 0.001). B-KPro = Boston keratoprosthesis; IOP = intraocular pressure; mi-KPro = minimally invasive keratoprosthesis; PKP = penetrating keratoplasty.

In our study, we demonstrate that the mi-KPro eyes exhibit stable over time postoperative IOP (13.3 ± 4.2 mmHg, range 9–24 mmHg) compared with baseline (15.6 ± 1.5 mmHg, range 13–19 mmHg; *P* = 0.138), as shown in (Fig 9A, C, E), with last follow-up of 15.5 ± 2.2 mmHg. Conversely, PKP eyes exhibit acute IOP elevations from 16.5 ± 2 mmHg to 25.8 ± 9.7 mmHg within 1 month (*P* = 0.003, n = 12), as depicted in Fig 9A, D, F. Notably, these PKP eyes do not respond to timolol eye drops (21.5 ± 10.5 mmHg). Similarly, B-KPro eyes exhibit marked IOP elevation from 17.6 ± 2.9 mmHg to 30 ± 11 mmHg within 1 week (*P* = 0.018, n = 6), which is effectively controlled by administering timolol 1-4 times daily for 3 weeks (18 ± 7 mmHg) as assessed clinically, as shown in (Fig 9B). Tonopen measurements show strong correlation with ground truth manometric measurements (*r* = 0.95, *P* = 0.001) as illustrated (Fig 9G, H).

## Discussion

Artificial corneas are an indispensable option for patients in whom conventional corneal transplantation is unlikely to succeed, yet the long-term success of currently available KPros remains constrained by glaucoma, infectious endophthalmitis, RPM formation, and retinal detachment.^10^, ^11^, ^12^ Clinical and experimental data suggest that these complications arise from a combination of extensive surgical trauma, chronic disruption of the corneal barrier function around the KPro optical stem, and persistent tissue inflammation that diffuses posteriorly.^17^^,^^18^^,^^23^, ^24^, ^25^, ^26^ In this study, we describe a minimally invasive keratoprosthesis (mi-KPro), specifically engineered to reduce surgical trauma, preserve aqueous outflow, and protect the posterior segment. We demonstrate favorable anatomic and functional outcomes compared with PKP and B-KPro in severe chemical injury models.

A central design feature of mi-KPro is the ultrathin, ultraflexible 70-μm nitinol backplate. Nitinol has an extensive clinical track record in cardiovascular and glaucoma implants owing to its biocompatibility, corrosion resistance, and superelastic behavior.^13^, ^14^, ^15^^,^^27^ Finite element analysis confirmed that the backplate tolerates physiologic intraocular loads with a safety factor well above unity and without exceeding the yield strength of the alloy. The thin, flexible backplate conforms to corneal curvature and distributes stress away from the limbus, while its open haptic architecture is expected to permit nutrient and fluid exchange between the aqueous humor and donor cornea. Although our simulations assumed linear elastic behavior, which underestimates the full superelastic response of nitinol, they provided a conservative assessment of mechanical safety; future models incorporating shape-memory/super-elastic behavior could further refine design parameters. By allowing controlled corneal deformation and micromotion, the backplate may support physiologic stromal remodeling and aqueous outflow, consistent with emerging data on mechano-metabolic coupling in ocular tissues.^28^, ^29^, ^30^, ^31^, ^32^

The modified DALK-based implantation strategy represents a second key element of the mi-KPro concept. By leaving a thin stromal layer and trephining only a 2.5-mm opening in Descemet membrane, the procedure avoids full-thickness corneal penetration and minimizes limbal trauma compared with PKP or B-KPro surgery.^21^^,^^33^, ^34^, ^35^, ^36^ In our models, mi-KPro implantation was technically straightforward for surgeons experienced with DALK, required operative times comparable to PKP, and resulted in minimal early postoperative inflammation. Importantly, the small Descemet’s opening prevented catastrophic sequelae, such as retinal detachment or endophthalmitis in eyes with device exposure or extrusion, suggesting that complications with mi-KPro may be more localized and manageable than with conventional B-KPro designs. Indeed, none of these eyes developed hypotony or retinal detachment after device extrusion, while the opening on the cornea self-sealed, underscoring the safety margin of the minimally invasive approach.

Infection and RPM formation remain major causes of morbidity after B-KPro implantation and are key drivers of the requirement for lifelong antimicrobial prophylaxis.^37^, ^38^, ^39^, ^40^ Despite using severe alkali and acid injury models—conditions known for intense ocular surface inflammation and frequent PKP failure^21^^,^^33^, ^34^, ^35^—we observed no long-term infections in mi-KPro eyes and absence of a membrane formation posterior to or encroaching upon the posterior optic of the mi-KPro (i.e., no visually obscuring RPM) over 12 months. The nitinol backplate and titanium sleeve appear to support rapid tissue integration, as evidenced by the fibrous tissue layer deposition, assessed with scanning electron microscopy, which likely reinforces the barrier against microbial ingress. The presence of localized stromal fibrous tissue around the intralamellar backplate is considered as a part of the corneal wound-healing response rather than a classic RPM. Beyond the typical antimicrobial prophylaxis, as recommended for B-KPro patients, periodic application of dilute povidone–iodine could represent a low-cost adjunctive strategy to reduce bioburden in high-risk corneas.^41^, ^42^, ^43^ As shown in this study, mi-KPro’s reduced reliance on daily topical antibiotics may be particularly advantageous for patients in resource-limited settings.

Chemical injuries to the cornea lead to retinal damage primarily by release of tumor necrosis factor alpha (TNF-α) that rapidly diffuses posteriorly with sustained effect for >3 months.^18^^,^^44^ This TNF-α–mediated inflammatory injury is distinct from, though ultimately linked to, secondary mechanisms involving microglia and monocyte-derived cells that contribute to longer-term neuroglial remodeling.^24^^,^^25^^,^^45^ In the present study, the injured host cornea was surgically excised 1 month after the burn and replaced with a healthy donor cornea, with or without a KPro, thereby markedly reducing the ongoing source of TNF-α, even though some postoperative inflammation inevitably remained. Consistent with our earlier findings,^17^^,^^25^^,^^18^^,^^22^^,^^45^ chemically injured eyes still exhibited retinal and ON degeneration relative to uninjured controls, but this damage was reduced in mi-KPro eyes compared with PKP-treated eyes, likely reflecting better IOP control, angle preservation, and reduced chronic inflammation. A future study should explore the effect of combining mi-KPro implantation with targeted anti–TNF-α therapy in mitigating retinal degeneration after severe chemical injury.

Glaucoma and optic neuropathy are among the most devastating complications after KPro surgery and are often difficult to detect because IOP cannot be measured accurately through a rigid backplate.^11^^,^^22^^,^^36^^,^^46^ Currently, IOP assessment in KPro patients relies largely on digital palpation, which is imprecise and operator-dependent.^46^ The mi-KPro engineering allows tonometry over the flexible backplate. In our study, TonoPen measurements in mi-KPro eyes correlated strongly with intracameral manometry (*r* = 0.95, *P* = 0.001). Intraocular pressure in mi-KPro eyes remained stable over time and did not change compared with baseline. No mi-KPro eye required antiglaucoma medication to achieve this. In contrast, PKP and B-KPro eyes showed early, sustained IOP elevation, which led to significant retinal thinning and optic nerve axon loss. The use of general anesthesia may lower IOP and should be considered when interpreting absolute IOP values. In this study, standardized conditions in IOP evaluation have partially mitigated this problem and its impact on comparative conclusions. Our data support the concept that limiting the surgical trauma and maintaining angle anatomy can mitigate the IOP elevation, inflammation, and subsequent neurodegeneration, as often observed after penetrating corneal surgery, including keratoprosthesis.^17^^,^^18^^,^^22^^,^^24^^,^^25^^,^^36^^,^^47^

Anatomic retention is another critical determinant of keratoprosthesis success. Penetrating keratoplasty grafts and B-KPro devices failed within approximately 3 months, consistent with the known poor prognosis of full thickness penetrating surgery.^21^^,^^33^, ^34^, ^35^ The initial mi-KPro backplate design (v1.0) achieved a 71.6% 12-month retention in alkali burns, and failures were associated with low contact area and tissue thinning over the elongated backplate haptics. Guided by finite element analysis, we revised the backplate architecture by increasing the haptic area, which resulted in 100% anatomic retention over 12 months without corneal thinning in a subsequent study with acid burns. Improved device retention with the mi-KPro v2.0 was likely related to focused backplate design optimizations and not to differences in the injury model. Future studies, especially in humans, would help evaluate the impact of this modification in clinical reality.

An important practical limitation of mi-KPro is its reliance on a modified DALK technique, which may be challenging in some cases with extremely scarred or previously grafted corneas. In our hands, the modified DALK achieved almost 100% success rate. The surgery allowed a residual corneal stroma of ∼100 μm over the Descemet membrane, which helped central 2.5 mm trephination for insertion of the mi-KPro optical stem into the anterior chamber. One surgery resulted in Descemetorexis, and the mi-KPro was implanted conventionally, using a full-thickness of 8.5 mm trephination. Interestingly, even as a penetrating device, the mi-KPro exhibited anatomic retention for 12 months without RPM, infection, or IOP elevation. The possibility of implanting the mi-KPro device using conventional techniques provides surgical flexibility for future human use.

The mi-KPro shares several fundamental constraints with current keratoprosthesis designs. Firstly, there is no true biological integration between the polymer/metal components and host tissue, and long-term fixation continues to rely on mechanical design and stromal wound healing rather than genuine biointegration. Although porous and tissue in-growth-promoting materials are under investigation, no such keratoprosthesis has yet received US Food and Drug Administration clearance. Secondly, mi-KPro requires a donor corneal, and therefore does not directly solve the problem of global donor tissue scarcity. We note, however, that KPro implantation is typically an elective, highly selected procedure, and in many regions, donor corneas can be procured through international eye bank networks, making logistics challenging but not prohibitive.

In summary, mi-KPro integrates a superelastic nitinol backplate with a minimally invasive implantation strategy to address several major failure modes of existing KPros. In severe chemical burn models, mi-KPro provided high anatomic retention, stable IOP, preservation of retinal and optic nerve structure, and low rates of infection and RPM formation compared with PKP and B-KPro. These findings support further development and clinical translation of mi-KPro as a next-generation keratoprosthesis for patients with end-stage corneal disease who are poor candidates for conventional transplantation.
