# Supplementary material for: Intermediate Term Results of a Novel Minimally Invasive Keratoprosthesis
Source: Ophthalmol Sci. 2026 Feb 18;6(4):101117. doi: 10.1016/j.xops.2026.101117 (PMC13011035; doi:10.1016/j.xops.2026.101117)
Supplement: Supplementary Figure S1 [file mmc1.pdf]

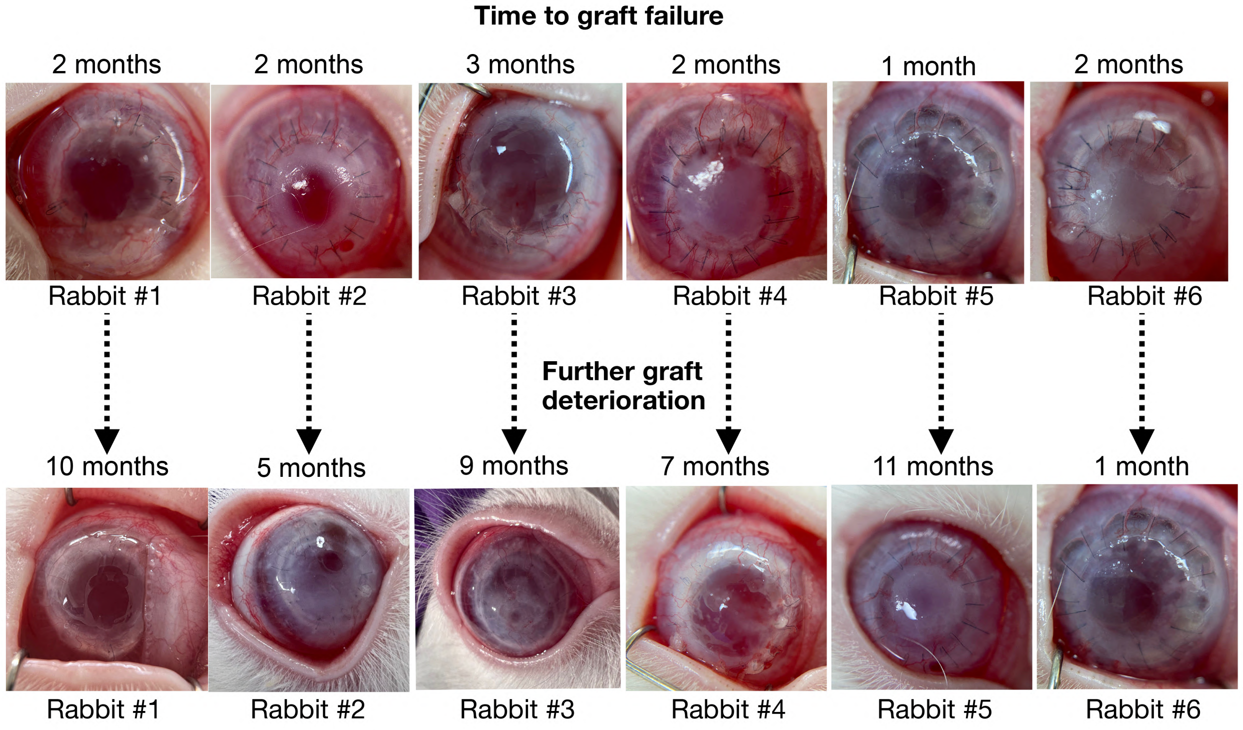

Supplementary Figure 1. Graft failure in PKP-treated eyes after alkali injury

a, Graft failure associated with corneal edema and neovascularization in alkali burned eyes. **b**, Further deterioration of the quality of the corneal graft over time.
