# Supplementary material for: Intermediate Term Results of a Novel Minimally Invasive Keratoprosthesis
Source: Ophthalmol Sci. 2026 Feb 18;6(4):101117. doi: 10.1016/j.xops.2026.101117 (PMC13011035; doi:10.1016/j.xops.2026.101117)
Supplement: Supplementary Figure S2 [file mmc2.pdf]

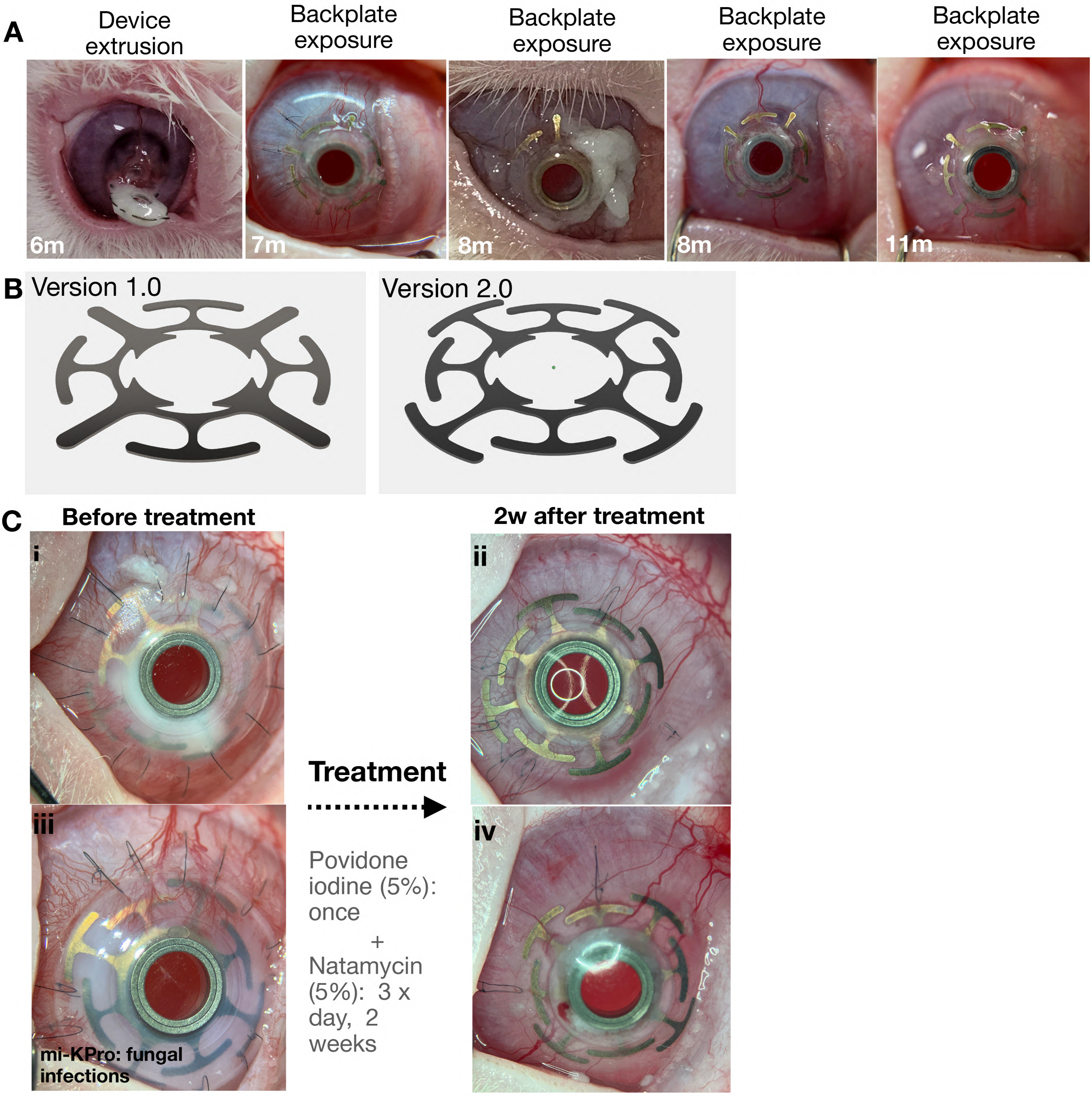

Supplementary Figure 2. Complications and management in mi-KPro

a, b, Earlier version (Version 1.0) of the mi-KPro in a rabbit eye with corneal alkali burn exhibiting either partial backplate exposure or extrusion. **b,** Redesigning the mi-KPro backplate to increased structural integrity and conformity to the donor tissue using finite element analysis, resulting in version 2.0. **c,** Two fungal infections in acid burns implanted with the mi-KPro version 2.0 due to donor tissue contamination. Both infections were successfully treated using 5% povidone-iodine and 5% natamycin eye drops.
