# Supplementary material for: Intermediate Term Results of a Novel Minimally Invasive Keratoprosthesis
Source: Ophthalmol Sci. 2026 Feb 18;6(4):101117. doi: 10.1016/j.xops.2026.101117 (PMC13011035; doi:10.1016/j.xops.2026.101117)
Supplement: Supplementary Figure S3 [file mmc3.pdf]

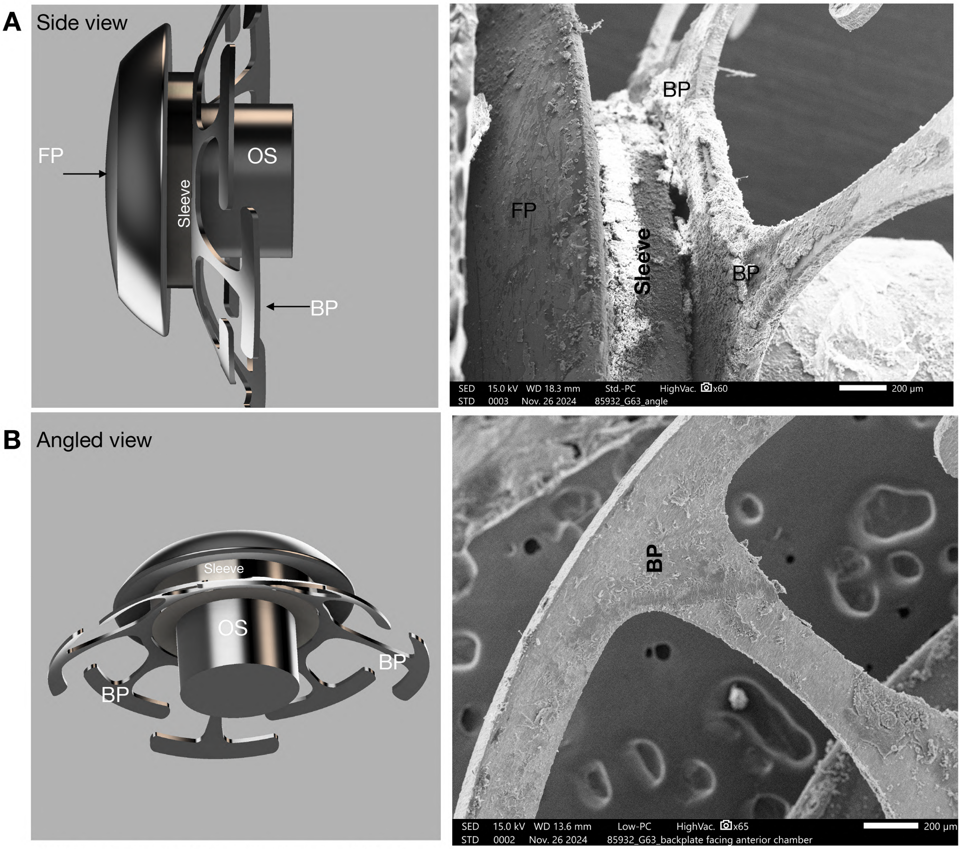

FP- front plate, OS- optical stem, BP- backplate

Supplementary Figure 3. Scanning electron microscopy of mi-KPro device integration

a, Side view demonstrating tissue attachment on the sleeve containing the donor corneal tissue.

b, Angled view illustrating tissue attachment to the haptics of the flexible backplate.
